# Ethanolic Extrusion of Indica Rice Flour for Rice Noodle Production

**DOI:** 10.3390/foods14091453

**Published:** 2025-04-23

**Authors:** Miaomiao Fu, Xing Zhou, Hong (Sabrina) Tian, Yanxin Chen, Zhengyu Jin

**Affiliations:** 1 State Key Laboratory of Food Science and Resources, Jiangnan University, Wuxi 214122, China; 13569178459@163.com (M.F.); zhouxing@jiangnan.edu.cn (X.Z.); 18356632392@163.com (Y.C.); 2 School of Food Science and Technology, Jiangnan University, Wuxi 214122, China; 3 China Collaborative Innovation Center of Food Safety and Quality Control in Jiangsu Province, Jiangnan University, Wuxi 214122, China; 4 School of Food Technology and Natural Sciences, College of Sciences, Massey University, Tennent Drive, Palmerston North 4410, New Zealand; h.tian@massey.ac.nz

**Keywords:** ethanolic extrusion, V-type indica rice flour, rice noodle premix, rice noodles

## Abstract

Due to the absence of gluten, rice noodles require complex processing to achieve a desirable texture. This study developed ethanolic-extruded indica rice flour (EERF) as a novel gluten substitute to simplify rice noodle production. EERF exhibited a distinct V-type crystalline structure (7.89% crystallinity) and high cold-paste viscosity (1043 cP), enabling its use as a binder in rice dough. When blended with native indica rice flour (IRF) at 10–20%, the EERF-IRF premix formed a cohesive dough with water via cold gelation, imparting viscoelasticity and tensile resistance. Optimal formulation (15% EERF for the premix and 37% water for making the dough) yielded fresh rice noodles with reduced cooking loss (5.57%) and a reduced breakage rate (14.44%), alongside enhanced sensory scores. This approach offers a clean-label, industrially scalable solution for producing rice noodles with simplified processing and improved quality.

## 1. Introduction

The growing demand for gluten-free products has intensified interest in improving rice-based alternatives, particularly rice noodles, which face inherent challenges due to the absence of gluten [1,2]. Unlike wheat noodles, which rely on gluten for viscoelasticity and structure [3], rice noodles require gelatinization and retrogradation of starch to form a stable gel network [4]. Traditional industrial methods for rice noodle production are laborious and time consuming and often yield products with compromised texture or high cooking loss. Furthermore, rice noodles are categorized by their moisture content—dry (<15%), semi-dry (15–60%), and wet (>60%)—each presenting trade-offs between shelf stability, cooking efficiency, and sensory quality [5]. Dry rice noodles, for instance, exhibit an extended shelf life but require prolonged cooking times, while wet rice noodles, although palatable, are prone to spoilage and structural deterioration [4,5].

To mimic gluten’s functional role, additives (summarized in Table 1) such as hydrocolloids (e.g., xanthan gum), proteins (e.g., zein and gliadin), enzymes (e.g., transglutaminase), and pregelatinized starch have been explored [6,7,8,9]. However, these solutions often demand high additive concentrations, costly processing steps, or result in undesirable sensory profiles. For example, protein-based binders require thermal activation above 40 °C to form networks, limiting their practicality, while pregelatinized starch necessitates large quantities due to low cold-paste viscosity [7,9]. Such limitations underscore the need for a clean-label, scalable approach to enhance rice dough cohesion without compromising nutritional or sensory attributes [10].

Recent advances in starch modification offer promising alternatives. Extrusion integrates feeding, heating, and molding with high throughput. These characteristics make it a cost-effective option for large-scale production. Zhou et al. [15] demonstrated that ethanolic extrusion could produce V-type cold-water-soluble starch (VCWSS) from corn starch, achieving cold-paste viscosity comparable to hot-gelatinized corn starch. This method leverages ethanol’s plasticizing effect on starch to create a porous, hydrophilic structure during extrusion and drying, enabling cold-water swelling. However, the impact of ethanolic extrusion on indica rice flour (IRF) (~80% starch, 9% protein, and 0.8% lipid) properties, particularly cold-paste viscosity and dough-forming capacity, remains unexplored.

This study aimed to develop ethanolic-extruded indica rice flour (EERF) as a gluten substitute for rice noodle production. We hypothesized that EERF’s unique physicochemical properties, including V-type crystallinity and high cold-paste viscosity, would enable cohesive dough formation via cold gelation. The objectives were to (1) characterize EERF’s functional properties, (2) optimize EERF-IRF blends for dough rheology and processability, and (3) evaluate the cooking and sensory qualities of resulting noodles. By addressing the limitations of existing methods, this work provides a novel, industrially viable strategy to streamline rice noodle production while aligning with clean-label trends.

## 2. Materials and Methods

### 2.1. Materials

Indica rice flour (IRF, starch content: 75.31 g/100 g; protein content: 8.57 g/100 g; fat content: 1.54 g/100 g; IRF’s D_10_, D_50_, D_90_, and D_(4,3)_ are 30.66 μm, 67.23 μm, 121.3 μm, and 72.43 μm, respectively) was purchased from Hunan Ju Bao Jin Hao Biotechnology Co., Ltd. (Xiangtan, China). Anhydrous ethanol (analytical grade, ≥0.997 purity) was purchased from Shanghai Titan Technology Co., Ltd. (Shanghai, China).

### 2.2. Preparation of EERF

**Figure 1:**
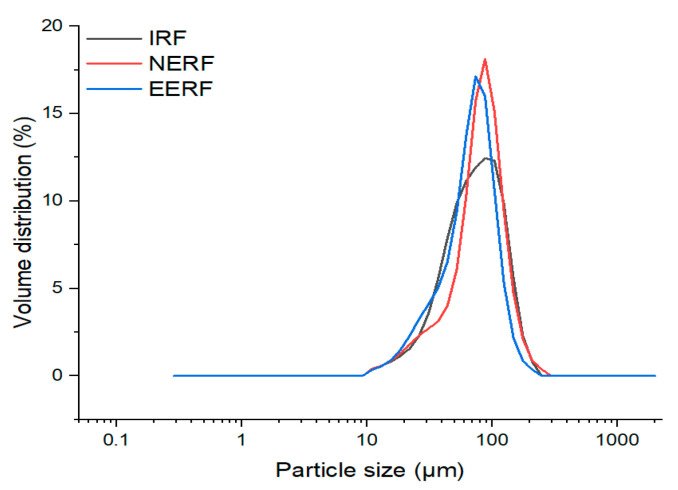
Particle size distributions of indica rice flour (IRF), no-ethanol extruded indica rice flour (NERF), and ethanolic-extruded indica rice flour (EERF).

Ethanolic-extruded indica rice flour (EERF) was prepared using a twin-screw extruder (POLYLAB-A, Thermo Fisher Scientific, Waltham, MA, USA) following the method described by Zhou et al. [15], with modifications. The experimental conditions taken for the preparation of EERF were as follows: The temperatures of the four temperature zones of the extruder were 55 °C, 65 °C, 120 °C, and 120 °C; the screw speed was 100 rpm; the concentration of ethanolic solution was 40% (*w/v*); and the ratio of IRF to 40% ethanol solution was 1:0.8 (*m/v*). Ethanol and water were premixed at a 40:60 volume ratio to obtain an ethanol solution, and during the extrusion process, the ethanol solution was continuously injected with a constant flow pump at a set flow rate. The die’s outer diameter, thickness, and hole diameter are 58, 15, and 3.5 mm, respectively. The extrudate was dried in an oven at 40 °C for 10 h. After that, it was crushed with a high-speed grinder (FW100, Tianjin Tester Instrument Co., Ltd., Tianjin, China), passed through a 100-mesh screen, and then stored in a sealed bag. The particle size distribution is shown in Figure 1. The no-ethanol extruded indica rice flour (NERF, control sample) was obtained by replacing the ethanol solution with water; the other operating conditions were kept the same.

### 2.3. Crystalline Structure

The determination was performed using an X-ray diffractometer (D2 Phaser, Bruker AXS GmbH, Karlsruhe, Germany). During the test, the samples were scanned from 3° to 36° at a scanning frequency of 3°/min. The tube voltage and tube current were kept at 40 kV, and 30 mA, respectively. The relative crystallinity was calculated using MDI JADE software (version 6.5, Materials Data, Inc., Livermore, CA, USA). The ratio (%) of the V-type peak area to the total dispersion area of the sample (4–30°) in the spectrum was the relative crystallinity [16].

### 2.4. Cold-Paste Viscosity

The cold-paste viscosity of the samples was determined using a Rapid Visco Analyzer (RVA, RVA 4500, Newport, Australia). Three grams of the sample and 25 mL of deionized water were mixed in an aluminum can, and the temperature of the entire test was kept at 25 °C. For the first 10 s, the speed of the stirring paddle was 960 rpm to disperse the sample fully, and then it was maintained at 160 rpm until the end of the experiment. The whole test process time was 5 min [17].

### 2.5. Hot-Paste Viscosity

The hot-paste viscosity of the samples was determined using a RVA. Three grams of the sample and 25 mL of deionized water were mixed in an aluminum can. The test procedures were as follows: equilibrating at 50 °C for 1 min, heating to 95 °C at 12 °C/min, holding at 95 °C for 2.5 min, cooling to 50 °C at 12 °C/min, and maintaining at 50 °C for 1.5 min. The impeller parameters are consistent with the cold-paste viscosity test.

### 2.6. Hydration Characteristics

Hydration characteristics refer to the water absorption index (WAI) and water solubility index (WSI). One gram of the sample and 20 mL of deionized water were mixed thoroughly in a 50 mL centrifuge tube that had been weighed. It was then incubated in a water shaker at 25 °C for 30 min. The sample after processing was centrifuged at 1444× *g* for 15 min to separate the supernatant from the precipitate, and then the supernatant was dried in a 110 °C oven to constant weight and weighed [18].

(1) $$WAI\left(\frac{g}{g}\right)=\frac{W_{2}}{W_{1}}$$

(2) $$WSI\left(\%\right)=\frac{W_{3}}{W_{1}}\times 100\%$$

W_1_ (g) is the weight of the dry sample, W_2_ (g) is the weight of the wet precipitate, and W_3_ (g) is the weight of the supernatant dried to a constant weight.

### 2.7. Water Absorption Properties

The water absorption properties of the rice noodle premix were measured using Mixolab (Chopin, Tripette et Renaud, Paris, France). The operating procedure used was Chopin S, the temperature was 30 °C, and the rotational speed was 80 rpm. The appropriate amount of water was set to achieve a consistency of 1.1 ± 0.05 Nm during the test [1].

### 2.8. Thermal Transition Properties

The thermal transition properties of IRF, NERF, EERF, and rice noodle premix were measured using differential scanning calorimetry (DSC, DSC7000, Seiko Instrument, Inc., Chiba, Japan). The sample (4 mg) and deionized water (12 μL) were added to the stainless-steel pan. After being sealed, it was stored in a refrigerator at 4 °C for 8 h to equilibrate the moisture. A blank stainless-steel pan was used as a reference. The samples were heated from 25 °C to 130 °C at a heating rate of 10 °C/min. The onset gelatinization temperature (T_o_), peak gelatinization temperature (T_p_), conclusion gelatinization temperature (T_c_), and enthalpy (ΔH) were calculated directly from the DSC curve.

### 2.9. Preparation of the Rice Noodle Premix

The rice noodle premix was made by thoroughly mixing rice flour and EERF. The proportions of EERF were 10%, 12.5%, 15%, 17.5%, and 20% based on pre-experiments. In the pre-experiments, when EERF was less than 10%, the rice dough was difficult to make, and when EERF was higher than 20%, the rice dough was too sticky to prepare. Rice dough could not be formed using the same amount (10–20%) of NERF.

### 2.10. Preparation of the Rice Noodles

To determine the effect of EERF content on the properties of the rice dough and rice noodles, the rice noodle premix (containing 10%, 12.5%, 15%, 17.5%, and 20% EERF) and water at a mass ratio of 64:36 (*w*/*w*) were kneaded into rice doughs. To determine the effect of water content on the properties of rice dough and rice noodles, rice noodle premix with an EERF content of 15% and different amounts of water (34%, 35%, 36%, 37%, and 38%) were made into rice doughs. The rice doughs were gradually rolled into sheets using a noodle machine (Model JMTD-168/140, Dongfu Co., Ltd., Beijing, China) until a thickness of 2 mm was achieved. Subsequently, the rice dough sheets were cut into fresh rice noodles with a width of 2.5 mm.

### 2.11. SEM of the Rice Doughs

The microstructure of the rice doughs was observed via scanning electron microscopy (SEM; Gemini 300, Zeiss, Oberkochen, Germany). Rice doughs with different EERF contents were made according to the method described in Section 2.9 and were freeze-dried before observation. The experiment was carried out at an accelerating voltage of 5 kV. The samples were observed at 1000× magnification using SEM [19].

### 2.12. Rheological Properties of the Rice Doughs

Rice dough was prepared according to the method described in Section 2.9. An aluminum parallel plate with a 40 mm diameter was selected. The rice dough was placed on the platform of the rheometer (DHR-3; TA Instruments, New Castle, DE, USA), the plate spacing was set to 2000 μm, the excess sample was scraped off, and the rheological determination was carried out after resting for 60 s. First, the amplitude was scanned: the frequency was set at 1 Hz, the change in the dynamic modulus in the range of 0.01–100% of the strain was detected, and the strain value of 0.1% was finally selected. The sample was scanned at a frequency of 0.1–100 Hz. The storage modulus (G′), the loss modulus (G″), and the loss tangent (tanδ) of the sample were recorded [20].

### 2.13. Texture Properties of the Rice Dough Sheets

The rice dough sheets were prepared with a thickness of 2 mm according to the method described in Section 2.9 and then cut into 4 cm diameter discs using a mold. The rice dough sheet discs were subsequently tested with a texture analyzer (TA.XTplus Physical Property Analyser: Stable Micro System, Godalming, UK). The probe used was P/36; the pretest, test, and post-test speeds were 1 mm/s; the trigger force was 5 g; and the compression ratio was 50%.

### 2.14. Tensile Strength Properties of the Fresh Rice Noodles

The tensile test of the fresh rice noodles was carried out by using the A/SPR probe, and the pretest, test, and post-test speeds were 1, 1, and 10 mm/s, respectively, with a trigger force of 5 g, and a stretching distance of 30 mm [21].

### 2.15. Properties of the Cooked Rice Noodles

#### 2.15.1. Water Absorption Rate Properties of the Cooked Rice Noodles

Five grams of fresh rice noodles were cooked in 0.5 L of boiling water until the white center turned transparent (optimal cooking time) and then rinsed with cold water for 30 s. They were weighed after drying the moisture on the surface with kitchen paper. The water absorption rate of the cooked rice noodles was calculated according to the following formula [22]:

(3) $$Water\;absorption\;rate\left(\%\right)=\frac{W_{1}-W_{2}}{W_{2}}\times 100\%$$

W_1_ (g) is the weight of cooked rice noodles, and W_2_ (g) is the weight of fresh rice noodles.

#### 2.15.2. Breakage Rate of the Cooked Rice Noodles

Thirty fresh rice noodles with a length of 15 cm were cooked until the optimal cooking time was reached. Then, the total number of rice noodles after cooking was counted. The breakage rate of the cooked rice noodles was calculated according to the following formula:

(4) $$Breakage\;rate\left(\%\right)=\frac{N-30}{30}$$

N is the total number of the rice noodles after being cooked.

#### 2.15.3. Cooking Loss of the Cooked Rice Noodles

Five grams of fresh rice noodles were cooked until the optimal cooking time was reached and then rinsed with cold water for 30 s. The rice noodles were transferred into an aluminum pan that had been dried to constant weight. They were then dried to a constant weight in an oven at 110 °C and weighed. To calculate the moisture content, 5 g of fresh rice noodles was weighed after they had been dried to a constant weight. The cooking loss of the cooked rice noodles was calculated according to the following formulas:

(5) $$Moisture\;content\left(\%\right)=\frac{W_{1}-W_{2}}{W_{1}}\times 100\%$$

(6) $$Cooking\;loss\left(\%\right)=\frac{W_{3}-W_{4}}{W_{1}\times (1-M)}\times 100\%$$

W_1_ (g) is the weight of fresh rice noodles, W_2_ (g) is the weight of fresh rice noodles after drying, W_3_ (g) is the weight of the cooked rice noodles and aluminum pan after drying, W_4_ (g) is the weight of the aluminum pan after drying, and M (%) is the moisture content of fresh rice noodles.

#### 2.15.4. Texture Properties of Cooked Rice Noodles

The rice noodles were cooked until the optimal cooking time was reached and then rinsed with cold water for 30 s before they were analyzed using TPA. The probe used was P/25, and the procedures were the same as those described in Section 2.13.

#### 2.15.5. Sensory Qualities of the Cooked Rice Noodles

The sensory evaluation and scoring criteria of the cooked rice noodles are shown in the Table 2. The fresh rice noodles were cooked until the optimal cooking time was reached, rinsed with cold water for 30 s, and then independently evaluated by eight testers trained in sensory evaluation.

### 2.16. Statistical Analysis

The experimental data were expressed as the mean ± standard deviation. SPSS 25.0 (SPSS Inc., Chicago, IL, USA) and Origin 2021 (Origin Lab Corporation, Northampton, MA, USA) were used for data analysis. One-way analysis of variance and Tukey’s test were used to verify significant differences between data (*p* < 0.05).

## 3. Results

### 3.1. Crystalline Structure

**Figure 2:**
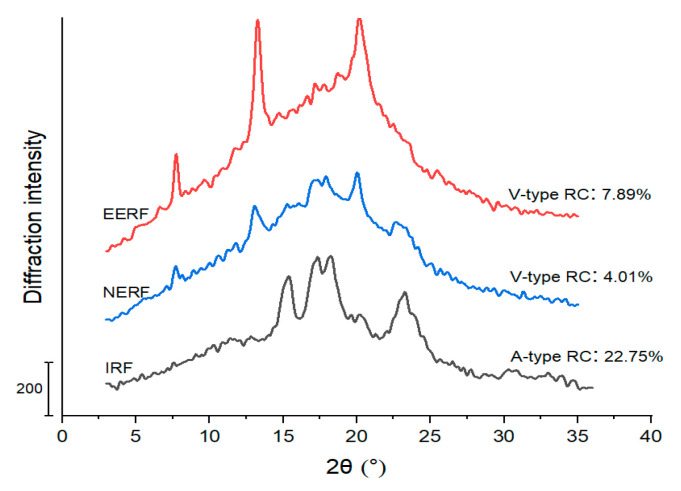
Crystalline structures of indica rice flour (IRF), ethanolic-extruded indica rice flour (EERF), and no-ethanol extruded indica rice flour (NERF). V-type RC: V-type relative crystallinity; A-type RC: A-type relative crystallinity.

Figure 2 shows that the IRF had an A-type crystalline structure, which is typical for native cereal starches [23]. After extrusion with water, new diffraction peaks appeared at 7°, 13°, and 21°, in the XRD profile, indicating that the NERF had an A+V-type crystalline structure [24]. The V-type crystalline structure was because of the formed amylose–lipid complex during extrusion [25]. However, the remaining A-type crystalline structure indicates that the NERF was not fully gelatinized under the extrusion conditions studied. The EERF showed a typical V-type crystalline structure that was more pronounced than that of the NERF. This is because not only the amylose–lipid complex but also the amylose–ethanol complex formed during the ethanolic extrusion [15].

### 3.2. Cold-Paste Viscosity

**Figure 3:**
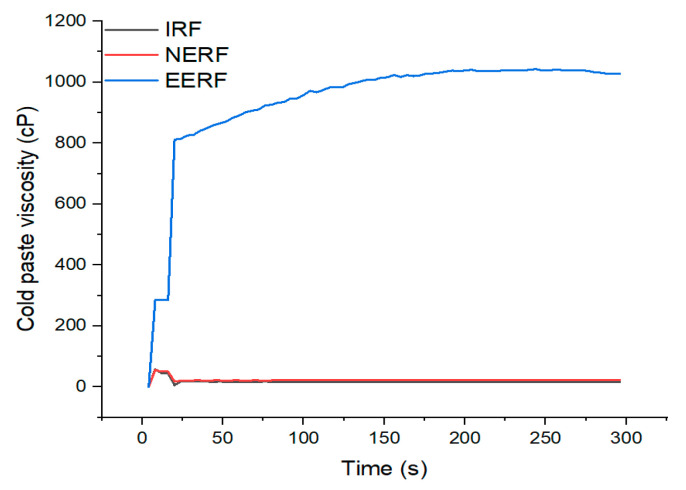
Cold-paste viscosity of indica rice flour (IRF), no-ethanol extruded indica rice flour (NERF), and ethanolic-extruded indica rice flour (EERF).

The cold-paste viscosities of IRF, NERF, and EERF are shown in Figure 3. It can be seen that the cold-paste viscosities of IRF and NERF were very low. Under the extrusion conditions explored in this study, the NERF was not fully gelatinized, and it could not swell well in cold water like the IRF. Zhou et al. [15] also found that extruded normal maize starch exhibited very low cold-paste viscosity. Meanwhile, the EERF showed a cold-paste viscosity of 1043 cP. This is because an amylose–ethanol complex formed during the ethanolic extrusion, and the ethanol during drying had evaporated and left V-type single-helical cavities, which had more hydrophilic hydroxyl groups exposed and could absorb more water when dissolved in cold water [19]. Additionally, the ethanolic extrusion process imparts a looser structure to EERF, facilitating greater water absorption [15]. Consequently, EERF’s high cold-paste viscosity endows it with the potential to act as a “gluten substitute” for rice noodle production.

### 3.3. Hot-Paste Viscosity

**Figure 4:**
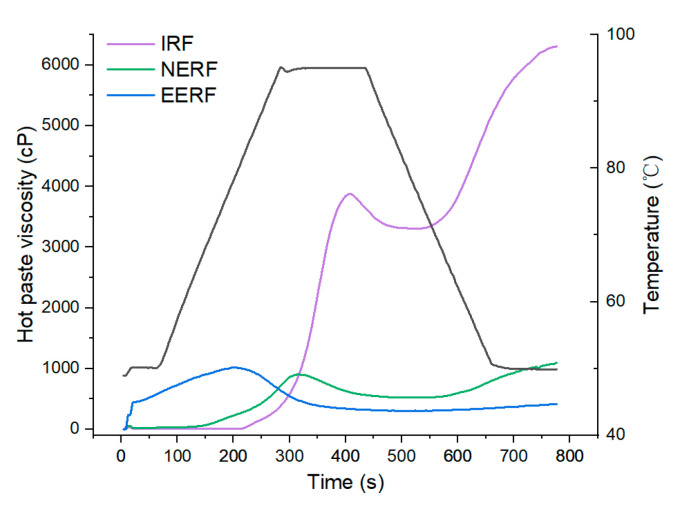
Hot-paste viscosity of indica rice flour (IRF), no-ethanol extruded indica rice flour (NERF), and ethanolic-extruded indica rice flour (EERF).

The hot-paste viscosities of IRF, NERF, and EERF are shown in Figure 4. Extrusion destroyed the starch granular structures, causing the denaturing of protein and degradation of starch in rice flour, thereby reducing the hot-paste viscosity [26,27]. Because gelatinization occurred during the extrusion process, the pasting temperatures of NERF and EERF reduced [15]. The EERF could absorb more water in cold water, so it readily pasted and had higher viscosity at low temperatures.

### 3.4. Hydration Characteristics

As shown in Table 3, the WAI of different rice flours increased after extrusion. Extrusion resulted in the exposure of hydrophilic hydroxyl groups, so the water absorption ability of the IRF after extrusion increased [28]. The EERF had more hydrophilic hydroxyl groups exposed, and the EERF became loose, and there were pores on the surface, which could combine with more cold water, so it had a higher WAI [15,29]. The WSI of the IRF increased after extrusion, and the WSI of the NERF was 3.230%, while that of the EERF was 4.495%. The high temperature and shear force during extrusion degraded the starch and other macromolecules in the IRF, producing water-soluble small molecules and, thus, increasing the WSI. However, for the ethanolic extrusion treatment, more soluble small molecules dissolved after water had entered the loose particles, resulting in a higher WSI for the EERF.

### 3.5. Water Absorption of the Rice Noodle Premix

**Figure 5:**
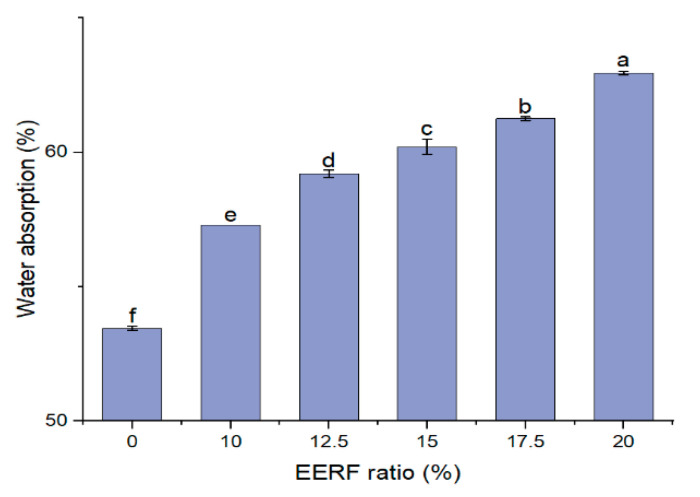
The water absorption of rice noodle premix with different ethanolic-extruded indica rice flour (EERF) contents. a–f: values with different lowercase letters are significantly different (*p* < 0.05).

As shown in Figure 5, the water absorption of the rice noodle premix became higher with an increase in the EERF content. After ethanolic extrusion, there were numerous hydrophilic hydroxyl groups exposed, which enhanced the ability of the EERF to bind water and, thus, resulted in increased water absorption [23]. Hence, when the EERF was added to the IRF, the water adsorption of the rice noodle premix increased.

### 3.6. Thermal Transition Properties of the Rice Noodle Premix

**Figure 6:**
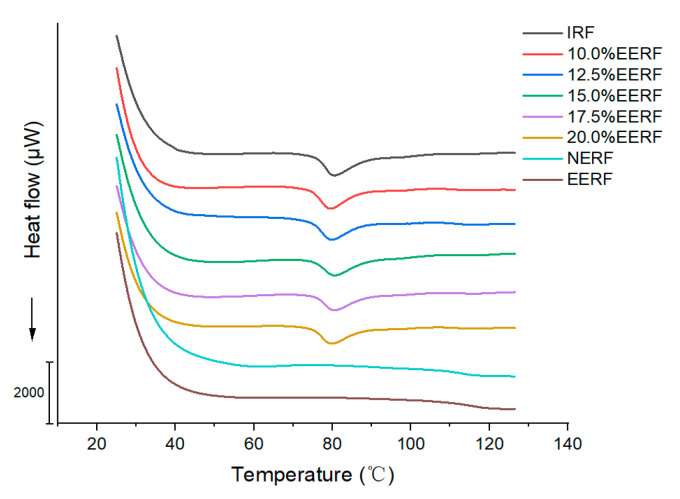
Thermal properties of indica rice flour (IRF), no-ethanol extruded indica rice flour (NERF), ethanolic-extruded indica rice flour (EERF), and rice noodle premix with different EERF contents.

The thermal transition properties of the IRF, NERF, EERF, and rice noodle premixed powder are shown in Figure 6 and Table 4. The gelatinization of the IRF occurred around 80 °C, and there was an obvious gelatinization endothermic peak. The gelatinization endothermic peak did not appear in the NERF or the EERF, which was due to the gelatinization of the NERF and EERF during the extrusion treatment. At around 120 °C, the extruded rice flour exhibited an endothermic melt peak, which was the melting peak for amylose–lipid complex [30]. No melting peak of the amylose–lipid complex was observed in the IRF, suggesting that the extrusion process promotes complex formation [31]. The V-type complexes of the NERF and EERF have similar melting enthalpy values, indicating that the V-type single helical cavities left by the V-type amylose–ethanol complex after drying disappeared after hydration with water. When the proportion of EERF in the rice noodle premix increased, the T_o_, T_p_, and T_c_ remained almost unchanged. This indicates that the addition of EERF did not affect the thermal stability of the rice noodle premix [32]. ΔH, which refers to the energy required to break the double helices in starch [33], decreased with an increasing EERF content. The ethanolic extrusion process melted the crystalline structure of starch so that when EERF was added to IRF, it led to a decrease in ΔH [34].

### 3.7. Scanning Electron Microscopy of Rice Doughs with Different EERF Contents

**Figure 7:**
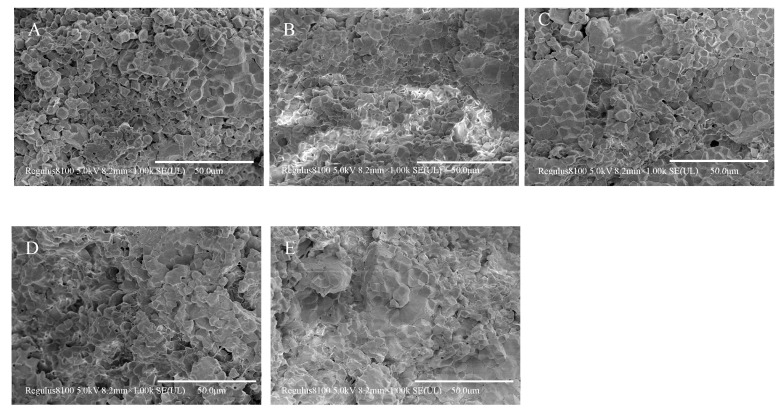
The scanning electron microscopy images of rice doughs. (**A**–**E**) were doughs prepared with rice noodle premix containing 10%, 12.5%, 15%, 17.5%, or 20% ethanolic-extruded indica rice flour (EERF) and a fixed water content of 36%, respectively.

The scanning electron microscopy (SEM) images of rice doughs with varying EERF contents are presented in Figure 7. Depending on the high cold-paste viscosity of EERF after hydration with water, the EERF can form a paste, which can bind the IRF and form the rice dough. When the EERF content in the rice noodle premix was 10.0%, the rice starch granules were largely separated from each other, with limited gel network formation. As the EERF proportion increased, the gel network structure gradually became denser, and the exposed rice flour particles became fewer.

### 3.8. Rheological Properties of the Rice Doughs

**Figure 8:**
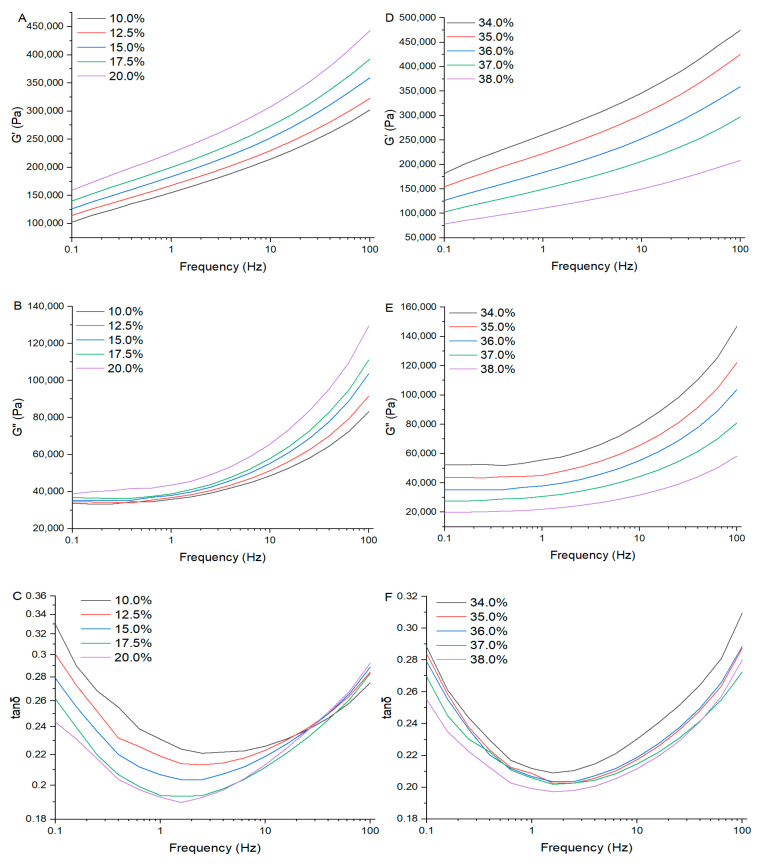
Rheological properties of the rice doughs. (**A**,**D**) Storage modulus (G′); (**B**,**E**) loss modulus (G″); (**C**,**F**) loss tangent (tanδ). (**A**–**C**) Dough prepared with rice noodle premix containing 10%, 12.5%, 15%, 17.5%, or 20% ethanolic-extruded indica rice flour (EERF) and a fixed water content of 36%. (**D**–**F**) Dough prepared with 15% EERF in the premixed powder and varying water content (34%, 35%, 36%, 37%, or 38%).

The rheological analysis results of the rice doughs are shown in Figure 8. In the scanning frequency range, the G′ of the rice doughs (Figure 8A,D) was always larger than the G″ (Figure 8B,E), and the tanδ (Figure 8C,F) of the rice doughs was less than 1, indicating that the rice doughs were mainly elastic [33]. When the moisture content was 36%, the G′ and G″ of the rice dough increased with an increased EERF proportion, and when the percentage of EERF increased to 20%, they also reached the maximum value, while the tanδ gradually decreased with an increased EERF addition. A smaller tanδ indicated a higher elastic ratio and a stronger network structure within the dough [35,36]. The results indicated that the gel formed by the EERF dissolved in cold water could give rice dough viscoelasticity [1]. With the increase in water, the G′, G″, and tanδ of rice doughs decreased, and when the percentage of water increased to 38%, they reached the minimum value (Figure 8). Previous studies also indicate that the dynamic modulus is affected by the moisture content: the higher the moisture content, the lower the value [37,38]. The moisture acted as an inert filler, causing the dynamic modulus of the rice doughs to change [37,39]. In the frequency sweep range, the G′ and G″ increased with an increasing frequency. These results indicate that the gels that formed after EERF hydration were frequency dependent. With the increase in EERF or water ratio, the rice doughs’ deformation ability increased because the gel network structure that had formed after EERF hydration became denser, or the increased moisture acted as a lubricant [1]. This is consistent with the SEM results.

### 3.9. Texture Properties of the Rice Dough Sheets

The texture analysis results of the rice dough sheets are shown in Table 5. When the water content was fixed at 36%, the hardness of rice dough sheets gradually decreased with the increased EERF proportion. This trend suggests that the paste formed after EERF hydration and its increased presence within the rice dough softens the overall structure. The adhesiveness of the rice dough sheets increased due to the high cold-paste viscosity of the EERF and the gradual increase in small soluble molecules. When analyzing the effect of water content, i.e., the EERF content was fixed at 15%, increasing the water proportion resulted in decreased hardness and increased adhesiveness of the rice noodle sheets. This change was due to the dilution effect of water, which softened the dough and enhanced the stickiness.

### 3.10. Tensile Strength Properties of the Fresh Rice Noodles

As shown in Table 5, with the increase in EERF content, the tensile force gradually increased, indicating that the cold paste formed after the hydration of the EERF gave the rice doughs stretch resistance [7]. With the increase in water content, the tensile resistance of the rice noodles gradually decreased. With the increase in moisture, the EERF cold paste was diluted, and its sticking power was reduced [21].

### 3.11. Properties of the Cooked Rice Noodles

#### 3.11.1. Cooking Qualities of the Cooked Rice Noodles

With the increase in EERF content (fixed the water content at 36%), the optimal cooking time first decreased and then increased (Table 6). The initial decrease in cooking time was attributed to the reduction in the ΔH of the rice noodle premix due to the addition of EERF. This reduction in ΔH lowered the heat energy required for cooking. However, when the EERF content increased to 17.5% and 20%, the cooking time rose again. This increase might be due to the formation of a denser gel network structure in the fresh rice noodles. As the EERF content rose beyond 15%, this denser network retarded water adsorption during cooking, making it more difficult for water to penetrate the noodles. It was supported by the fact that the water adsorption rate during cooking decreased with the increase in EERF content (Table 6). This behavior contrasted with the increased water absorption observed in the rice noodle premixed powder with higher EERF proportions, as shown in Figure 3. The water absorption capacity of the premixed powder primarily affected the water requirement during rice dough formation rather than the cooking process itself.

The cooking loss of rice noodles showed a tendency to first decrease and then increase with the increased content of EERF (Table 6). At low EERF addition, the loose gel network formation led to the loss of IRF during cooking. In contrast, at higher EERF addition, the water-soluble nature of the EERF and the longer cooking time resulted in more soluble small molecules being released, which contributed to increased cooking loss [17].

The breakage rate of rice noodles decreased with the increased content of EERF due to the formation of a denser network structure. This tighter structure enhanced the overall integrity of the noodles during cooking, thereby reducing breakage.

With the increase in water content (while fixing the EERF content at 15%), the optimal cooking time decreased. When the moisture content was low, the rice noodles required more time to absorb sufficient water for complete gelatinization, resulting in a longer cooking time. As the water content increased, the water absorption rate of the rice noodles gradually decreased. This is because the water absorption capacity of the 15% EERF rice noodle premixed powder is fixed; hence, as the initial moisture content of the raw rice dough increased, the additional water absorption needed during cooking decreased. Both the cooking loss and breakage rate increased with the increase in water content. This is attributed to the dilution effect, which gradually loosened the gel network structure of the noodles. As a result, the encapsulation of IRF within the gel network was reduced, leading to higher cooking loss and an increased breakage rate during cooking.

#### 3.11.2. Texture Qualities of the Cooked Rice Noodles

As shown in Table 7, with an increasing EERF proportion in the fresh rice noodles, the hardness, springiness, and chewiness of the cooked rice noodles decreased. During cooking, IRF, which was the major component of the rice noodles, gelatinized and primarily contributed to the texture properties. The high temperature and shear force during extrusion broke the intermolecular links of starch, reducing the gelling ability of the extruded starch [40]. Consequently, a higher EERF content resulted in a softer texture for the cooked rice noodles. With an increasing EERF proportion, the adhesiveness of the cooked rice noodles gradually decreased. At low EERF levels, the hydrated EERF was insufficient to strongly encapsulate IRF, leading to low tensile strength in the fresh rice noodles (as shown in Table 5). This insufficient encapsulation caused the dissolution of some small molecules during cooking, resulting in higher adhesiveness of the cooked rice noodles. In contrast, at higher EERF levels, there were no significant differences in adhesiveness because the denser gel network structure might compensate for the presence of more soluble small molecules.

With an increasing water content in the fresh rice noodles, the hardness and chewiness of the cooked rice noodles gradually decreased, while the adhesiveness first decreased and then increased, and the springiness gradually increased. When the moisture content was low, the cooked rice noodles required more time to absorb sufficient water during cooking. By the optimal cooking time, the internal IRF (Isolated IRF) was just gelatinized, whereas the external IRF was overcooked. This uneven gelatinization resulted in higher adhesiveness and lower springiness of the cooked rice noodles [41]. In contrast, when the water content in the fresh rice noodles was high, the overall gelatinization of the cooked rice noodles was more uniform. The external IRF achieved a more appropriate degree of cooking compared to noodles with a lower moisture content, resulting in higher springiness. However, the looser gel network structure in high-moisture noodles allowed small molecules to dissolve more easily, leading to increased adhesiveness.

#### 3.11.3. Sensory Scores of the Cooked Rice Noodles

The sensory scores of the cooked rice noodles prepared with different EERF contents or moisture levels are presented in Table 8. There were no significant differences in color and flavor among the cooked rice noodles prepared with varying amounts of EERF or water. When the moisture content was fixed at 36%, the highest sensory score was achieved when the EERF content in the premix was 15%. This suggests that rice noodles with appropriate hardness, low adhesiveness, and high elasticity are more readily accepted by consumers. Similarly, when the EERF content was fixed at 15%, the highest sensory score was obtained with a water content of 37%.

Therefore, the optimal preparation condition for rice noodles was 15% EERF for the premix and a premix-to-water ratio of 63:37 (*w*/*w*).

## 4. Conclusions

Ethanolic-extruded indica rice flour with a V-type crystalline structure and superior cold-paste viscosity was successfully prepared using an extruder. The cold gel formed by ethanolic-extruded indica rice flour hydration mimicked gluten functionality, enabling dough formation with enhanced elasticity and tensile strength. A premix containing 15% ethanolic-extruded indica rice flour together with 37% water produced rice noodles with optimal cooking properties (low breakage and minimal cooking loss) and good sensory attributes. This study demonstrates a practical, clean-label strategy to streamline rice noodle production, addressing industrial challenges associated with gluten-free formulations. The method’s efficiency and scalability highlight its potential for both home and commercial applications.

## Figures and Tables

**Table 1:** Recent advances in rice noodles with gluten substitutes.

Type of Additive	Additive	Mechanism of Action	Pros and Cons	References
Protein	The ratio of zein and rice flour is 5:95	The zein can form a viscoelastic network structure above the glass transition temperature	Good mixing stability; high cost, poor color sensory, and low extensibility, taking 6 min to form network structure above 40 °C	[9]
Enzyme	1.5% transglutaminase (TGase) of total weight of rice flour, wheat flour, and vital gluten; the ratio of rice flour, wheat flour, vital gluten, table salt, and guar gum is set at 80:10:10:2:1	TGase can catalyze the formation of covalent cross-linked bonds between protein molecules	Good sensory quality; takes more than 1 h to prepare; high gluten content	[11]
Hydrocolloid	2.5% xanthan gum of resistant rice starch; the ratio of resistant rice starch, salt, NaHCO_3_, and full egg is 100:3:3:30	Xanthan gum can form viscous solution, and the hydrogen bonds and starch of xanthan gum form a stable gluten-like structure	Gluten-free; fast preparation; high xanthan gum addition	[12]
Pregelatinized starch	The ratio of extrude pregelatinized starch, rice flour, NaCO_3_, and NaCl is 60:40:0.3:1	It has cold-paste viscosity	Gluten-free; high pregelatinized rice flour addition	[7]
Composite	The ratio of rice flour, rice protein isolate, and NaCl is 90:10:1 and 1% TGase	The rice protein isolate can catalyze the formation of covalent cross-linked bonds between protein molecules	Nutritious and gluten-free but expensive and takes more than 2 h to prepare	[13]
	The ratio of heat-treated glutinous rice flour and xanthan gum (95:5) and rice flour is 10:90	Xanthan gum polymers form cross-links between starch granules during heat treatment	Gluten-free, with low xanthan gum addition	[14]

**Table 2:** Grading criteria for the cooked rice noodles.

	Grading Criteria
Color (15 points)	whiter, uniform color (10–15)
	white, uniform color (5–10)
	yellow and uneven color distribution (0–5)
Flavor (10 points)	rich rice flavor with no peculiar smell (7–10)
	rice aroma and a slight peculiar smell (3–7)
	faint rice flavor and a peculiar smell (0–3)
Organizational morphology (15 points)	smooth surface, compact structure (10–15)
	less smooth surface, less compact structure (5–10)
	adhesive surface, loose structure (0–5)
Hardness (20 points)	moderate (14–20)
	harder or softer (7–14)
	too hard or too soft (0–7)
Slippery (20 points)	smoother, non-sticky (14–20)
	smooth, moderate viscosity (7–14)
	not slippery, sticky (0–7)
Elasticity (20 points)	good elasticity, chewiness (14–20)
	medium elasticity, slight dente (7–14)
	poor elasticity, poor chewiness (0–7)

**Table 3:** Hydration characteristics of indica rice flour (IRF), no-ethanol extruded indica rice flour (NERF), and ethanolic-extruded indica rice flour (EERF).

	IRF	NERF	EERF
WAI (g/g)	1.985 ± 0.054 ^c^	4.088 ± 0.018 ^b^	7.546 ± 0.069 ^a^
WSI (%)	0.465 ± 0.262 ^c^	3.230 ± 0.311 ^b^	4.495 ± 0.191 ^a^

Values with different lowercase letters are significantly different (*p* < 0.05).

**Table 4:** Thermal properties of rice noodle premix with different ethanolic-extruded indica rice flour (EERF) contents.

EERF Content	T_o_ (°C)	T_p_ (°C)	T_c_ (°C)	ΔH (J/g)
0.0%	77.45 ± 0.21 ^a^	80.35 ± 0.07 ^ab^	86.25 ± 0.21 ^a^	7.96 ± 0.20 ^a^
10.0%	77.60 ± 0.28 ^a^	80.30 ± 0.00 ^ab^	85.20 ± 0.57 ^b^	7.08 ± 0.38 ^b^
12.5%	77.45 ± 0.21 ^a^	80.20 ± 0.14 ^ab^	85.65 ± 0.21 ^ab^	6.76 ± 0.10 ^b^
15.0%	77.70 ± 0.14 ^a^	80.35 ± 0.35 ^ab^	85.65 ± 0.07 ^ab^	5.75 ± 0.04 ^c^
17.5%	77.70 ± 0.28 ^a^	80.55 ± 0.07 ^a^	85.75 ± 0.24 ^ab^	5.46 ± 0.14 ^cd^
20.0%	77.45 ± 0.23 ^a^	80.10 ± 0.14 ^b^	85.20 ± 0.57 ^b^	5.12 ± 0.04 ^d^

Values with different lowercase letters are significantly different (*p* < 0.05).

**Table 5:** Texture of the rice doughs and tensile strength of the fresh rice noodles.

		Hardness (N)	Adhesiveness (N.s)	Tensile Strength (N)
EERF con-tent in the premix	10.0%	96.176 ± 3.912 ^a^	−0.461 ± 0.080 ^a^	0.129 ± 0.010 ^d^
	12.5%	85.443 ± 3.248 ^b^	−0.636 ± 0.061 ^ab^	0.150 ± 0.009 ^cd^
	15.0%	71.215 ± 1.827 ^c^	−0.743 ± 0.089 ^b^	0.168 ± 0.009 ^bc^
	17.5%	62.750 ± 1.924 ^d^	−0.978 ± 0.101 ^c^	0.185 ± 0.005 ^ab^
	20.0%	51.842 ± 2.908 ^d^	−1.106 ± 0.094 ^c^	0.205 ± 0.013 ^a^
Water ratio for making the dough	34.0%	101.734 ± 5.212 ^a^	−0.425 ± 0.074 ^a^	0.216 ± 0.007 ^a^
	35.0%	89.560 ± 5.543 ^b^	−0.597 ± 0.070 ^ab^	0.193 ± 0.006 ^a^
	36.0%	71.215 ± 1.827 ^c^	−0.743 ± 0.089 ^b^	0.168 ± 0.009 ^b^
	37.0%	57.239 ± 3.486 ^d^	−1.492 ± 0.188 ^c^	0.142 ± 0.014 ^c^
	38.0%	44.758 ± 1.003 ^e^	−1.825 ± 0.091 ^d^	0.111 ± 0.009 ^d^

Values with different lowercase letters are significantly different (*p* < 0.05). EERF is ethanolic-extruded indica rice flour.

**Table 6:** The cooking qualities of cooked rice noodles.

		Cooking Time (min)	Water Absorption (%)	Breakage Rate (%)	Cooking Loss (%)
EERF con-tent in the premix	10.0%	5.83	98.58 ± 2.24 ^a^	16.67 ± 3.34 ^a^	6.48 ± 0.23 ^a^
	12.5%	5.75	91.32 ± 1.05 ^b^	13.33 ± 3.34 ^ab^	5.60 ± 0.27 ^b^
	15.0%	5.58	86.74 ± 0.93 ^c^	12.22 ± 1.92 ^ab^	4.63 ± 0.28 ^c^
	17.5%	5.75	83.18 ± 0.40 ^cd^	10.00 ± 3.33 ^b^	5.35 ± 0.12 ^b^
	20.0%	5.83	79.17 ± 2.55 ^d^	7.78 ± 1.92 ^b^	5.79 ± 0.19 ^b^
Water ratio for making the dough	34.0%	6.17	95.18 ± 1.51 ^a^	6.67 ± 3.34 ^a^	3.64 ± 0.18 ^d^
	35.0%	5.92	91.05 ± 2.06 ^b^	8.89 ± 1.92 ^ab^	4.08 ± 0.22 ^d^
	36.0%	5.58	86.74 ± 0.93 ^c^	12.22 ± 1.92 ^bc^	4.63 ± 0.28 ^c^
	37.0%	5.33	83.03 ± 1.09 ^c^	14.44 ± 1.93 ^cd^	5.57 ± 0.16 ^b^
	38.0%	5.08	77.70 ± 1.37 ^d^	17.78 ± 1.92 ^d^	6.40 ± 0.16 ^a^

Values with different lowercase letters are significantly different (*p* < 0.05). EERF is ethanolic-extruded indica rice flour.

**Table 7:** Texture qualities of the cooked rice noodles.

		Hardness (g)	Adhesiveness (g.s)	Springiness	Chewiness (g)
EERF content in the premix	10.0%	973.804 ± 17.588 ^a^	−35.020 ± 1.607 ^a^	0.917 ± 0.008 ^a^	605.529 ± 21.509 ^a^
	12.5%	927.769 ± 12.180 ^b^	−29.527 ± 2.098 ^b^	0.904 ± 0.002 ^b^	579.382 ± 13.310 ^ab^
	15.0%	884.966 ± 21.978 ^c^	−25.303 ± 1.276 ^b^	0.896 ± 0.004 ^b^	545.595 ± 20.842 ^bc^
	17.5%	850.564 ± 9.518 ^c^	−25.391 ± 2.261 ^b^	0.874 ± 0.005 ^c^	506.547 ± 8.589 ^c^
	20.0%	808.914 ± 11.921 ^d^	−26.707 ± 1.248 ^b^	0.860 ± 0.011 ^d^	460.475 ± 16.351 ^d^
Water ratio for making the dough	34.0%	1008.997 ± 25.340 ^a^	−29.541 ± 1.416 ^b^	0.863 ± 0.004 ^d^	636.378 ± 14.344 ^a^
	35.0%	955.732 ± 29.138 ^a^	−26.750 ± 1.016 ^ab^	0.880 ± 0.009 ^c^	582.063 ± 11.356 ^b^
	36.0%	884.966 ± 21.978 ^b^	−25.303 ± 1.276 ^a^	0.896 ± 0.004 ^b^	545.595 ± 20.842 ^b^
	37.0%	825.090 ± 10.734 ^c^	−26.002 ± 0.659 ^ab^	0.905 ± 0.006 ^ab^	487.473 ± 11.729 ^c^
	38.0%	744.368 ± 17.857 ^d^	−30.042 ± 2.648 ^b^	0.912 ± 0.003 ^a^	453.126 ± 22.639 ^c^

Values with different lowercase letters are significantly different (*p* < 0.05). EERF is ethanolic-extruded indica rice flour.

**Table 8:** Sensory scores of the rice noodles.

		Color	Flavor	Organizational Morphology	Hardness	Slippery	Elasticity	Total Score
EERF con-tent in the premix	10.0%	13.75 ± 0.89 ^a^	9.50 ± 0.76 ^a^	10.13 ± 1.13 ^b^	12.50 ± 0.93 ^c^	13.25 ± 0.71 ^b^	14.50 ± 1.19 ^a^	73.63 ± 1.06 ^c^
	12.5%	13.00 ± 1.07 ^ab^	9.13 ± 0.64 ^ab^	11.38 ± 1.30 ^a^	13.25 ± 1.04 c	14.13 ± 0.83 ^ab^	13.88 ± 0.83 ^a^	74.75 ± 1.16 ^b^
	15.0%	12.63 ± 0.74 ^b^	8.88 ± 0.83 ^ab^	12.38 ± 0.92 ^a^	15.25 ± 0.89 ^ab^	14.75 ± 1.04 ^a^	12.50 ± 0.76 ^b^	76.38 ± 0.74 ^a^
	17.5%	12.25 ± 1.04 ^b^	8.75 ± 0.71 ^ab^	12.13 ± 1.36 ^a^	15.75 ± 0.89 ^a^	13.75 ± 1.28 ^ab^	11.63 ± 1.30 ^b^	74.25 ± 0.89 ^ab^
	20.0%	12.00 ± 1.07 ^b^	8.63 ± 0.74 ^b^	11.50 ± 1.20 ^a^	14.50 ± 1.41 ^b^	13.88 ± 0.83 ^ab^	12.25 ± 1.16 ^b^	72.75 ± 1.83 ^ab^
Water ratio for making the dough	34.0%	12.50 ± 1.20 ^a^	9.25 ± 0.71 ^a^	12.13 ± 0.83 ^ab^	13.25 ± 0.71 ^b^	13.75 ± 1.89 ^b^	10.88 ± 1.73 ^c^	72.25 ± 0.89 ^d^
	35.0%	12.25 ± 0.71 ^a^	8.75 ± 0.89 ^a^	12.88 ± 1.13 ^a^	14.38 ± 1.30 ^ab^	14.24 ± 1.16 ^b^	11.38 ± 1.06 ^c^	73.88 ± 1.13 ^c^
	36.0%	12.63 ± 0.74 ^a^	8.88 ± 0.83 ^a^	12.38 ± 0.92 ^a^	15.25 ± 0.89 ^a^	14.75 ± 1.04 ^ab^	12.50 ± 0.76 ^b^	76.38 ± 0.74 ^b^
	37.0%	13.25 ± 0.71 ^a^	9.38 ± 0.74 ^a^	11.13 ± 0.83 ^bc^	14.88 ± 1.36 ^ab^	15.38 ± 1.06 ^a^	14.13 ± 0.99 ^a^	78.13 ± 1.55 ^a^
	38.0%	12.88 ± 0.64 ^a^	9.00 ± 1.25 ^a^	10.63 ± 1.60 ^c^	13.75 ± 1.28 ^b^	14.38 ± 1.06 ^ab^	14.75 ± 1.04 ^a^	75.38 ± 1.77 ^b^

Values with different lowercase letters are significantly different (*p* < 0.05). EERF is ethanolic-extruded indica rice flour.

## Data Availability

The original contributions presented in the study are included in the article. Further inquiries can be directed to the corresponding author.

## Abbreviations

The following abbreviations are used in this manuscript:

EERF	ethanolic-extruded indica rice flour
IRF	indica rice flour
VCWSS	V-type cold water-soluble starch
NERF	no-ethanol extruded indica rice flour
RVA	Rapid Visco Analyzer
WAI	water absorption index
WSI	water solubility index
DSC	differential scanning calorimetry
SEM	scanning electron microscope
T_o_	onset gelatinization temperature
T_p_	peak gelatinization temperature
T_c_	conclusion gelatinization temperature
ΔH	enthalpy
G′	storage modulus
G″	loss modulus
tanδ	loss tangent
V-type RC	V-type relative crystallinity
A-type RC	A-type relative crystallinity
